# Supplementary material for: Lycosin-II Exhibits Antifungal Activity and Inhibits Dual-Species Biofilm by Candida albicans and Staphylococcus aureus
Source: J Fungi (Basel). 2022 Aug 24;8(9):901. doi: 10.3390/jof8090901 (PMC9504746; doi:10.3390/jof8090901)
Supplement: Supplementary file 1 [file jof-08-00901-s001.zip › jof-1831281-supplementary.pdf]

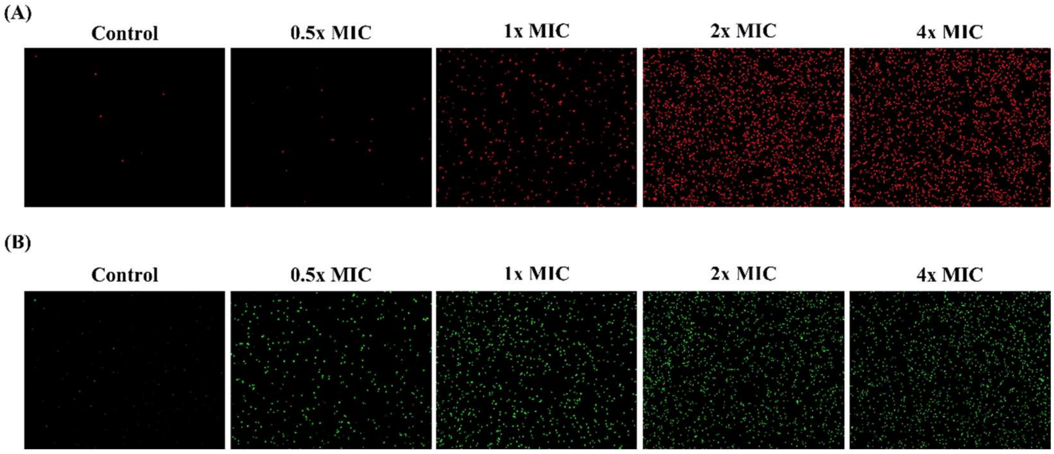

Figure S1. Fluorescence microscopy of C. albicans stained (A) PI and (B) SYTOX Green after treatement with Lycosin-II at 0.5×, 1×, 2× and 4× MIC. Theses images were obtained by using an EVOS FL Auto 2 imaging system. C. albicans without treatment was used as controls.
